# Depressive and Anxiety Symptoms of Healthcare Workers in Intensive Care Unit Under the COVID-19 Epidemic: An Online Cross-Sectional Study in China

**DOI:** 10.3389/fpubh.2021.603273

**Published:** 2021-03-05

**Authors:** Xiaofan Peng, Xiangyu Meng, Li Li, Chenghuan Hu, Wei Liu, Zhiyong Liu, Xinhua Ma, Daomiao Xu, Zhenhua Xing, Zhaowei Zhu, Bangshan Liu, Lina Zhang, Milin Peng

**Affiliations:** ^1^Department of Critical Care Medicine, Xiangya Hospital, Central South University, Changsha, China; ^2^Department of Urology, Zhongnan Hospital, Wuhan University, Wuhan, China; ^3^Department of Emergency Medicine, The Second Xiangya Hospital, Central South University, Changsha, China; ^4^Department of Cardiology, The Second Xiangya Hospital, Central South University, Changsha, China; ^5^Department of Psychiatry, The Second Xiangya Hospital, Central South University, Changsha, China

**Keywords:** COVID-19, ICU, mental health, depression, anxiety

## Abstract

**Background:** Since the coronavirus disease-2019 (COVID-19) outbreak, intensive care unit (ICU) healthcare workers were responsible for the critical infected patients. However, few studies focused on the mental health of ICU healthcare workers. This study aimed to investigate the psychological impact of COVID-19 on ICU healthcare workers in China.

**Methods:** We distributed the nine-item Patient Health Questionnaire (PHQ-9) and seven-item General Anxiety Disorder questionnaire (GAD-7) online to ICU healthcare workers in China. Respondents were divided into frontline and second-line according to whether they have contact with COVID-19 patients. Depressive and anxiety symptoms of all respondents were evaluated based on their questionnaire scores.

**Results:** There were 731 ICU healthcare workers finally enrolled in our study, including 303 (41.5%) male, 383 (52.4%) doctors, and 617 (84.4%) aged 26–45 years. All in all, 482 (65.9%) ICU healthcare workers reported symptoms of depression, while 429 (58.7%) reported anxiety. There was no significant difference between frontline (*n* = 325) and second-line (*n* = 406) respondents in depression (*P* = 0.15) and anxiety severity (*P* = 0.56). Logistic regression analysis showed that being female, ICU work time >5 years, and night duty number ≥10 were risk factors of developing depressive and anxiety symptoms. Income reduction was separately identified as risk of anxiety. Additionally, ICU work time >5 years was also identified as risk of developing moderate–severe depressive and anxiety symptoms.

**Conclusions:** Frontline ICU work was not associated with higher risk of depressive and anxiety symptoms during COVID-19 pandemic remission period in China. Actions like controlling night duty number, ensuring vacation, and increasing income should be taken to relieve mental health problem. Furthermore, we should pay close attention to those who had worked long years in ICU.

## Introduction

In the winter of 2019, a novel coronavirus disease (coronavirus disease-2019, COVID-19) was first reported in Hubei province of China and gradually developed a global public health crisis; over 26 million people have been diagnosed with COVID-19 infection, and nearly 1 million died at the beginning of this in September. The COVID-19 was the third coronavirus disease in the 21st century after severe acute respiratory syndrome (SARS) and Middle East respiratory syndrome (MERS); although the three coronavirus diseases shared similar transmission routes mainly by droplet and direct contact spread, the COVID-19 showed much higher infectivity (*R*_0_, 2.0–2.68) than SARS (*R*_0_, 1.7–1.9) and MERS (*R*_0_ <1) (1– 4). COVID-19 had a relatively long median incubation period of 5 days, and up to 50% patients with positive test results could be asymptomatic, which had been proved to be infectious (5–7). Moreover, COVID-19 caused a lower mortality rate of 2.2–3.5% compared to 9.5% for SARS and 34.4% for MERS, which led to less attention both from general population and certain governments (1, 8, 9). All these factors most likely contributed to the transmission of COVID-19. In the process of fighting COVID-19, intensive care unit (ICU) healthcare workers were responsible for the most critically infected patients, and many routine procedures in ICU including intubation, sputum aspiration, or use of nebulizers could be high risk due to the possible aerosol transmission (10). Furthermore, shortage of medical materials, work overload, and up to 50% mortality among severe infection patients in ICU bring mental burden to ICU healthcare workers (11). The experience of battling SARS had already shown adverse psychological impact on medical staff (12). During the COVID-19 pandemic, Kang's study also revealed a 28.6% moderate–severe mental health disturbance among 994 medical workers in Wuhan (13). Similarly, Lai's study reported symptoms of depression for 50.4%, anxiety for 44.6%, and insomnia for 34.0% among 1,021 medical workers in Hubei province and 236 outside Hubei province (14). All these information alerted more attention to mental health of medical workers. However, there were few mental health surveys specially designed for ICU healthcare workers. In order to find out the psychological effects of COVID-19 on ICU healthcare workers in China, we conducted this study.

## Materials and Methods

### Study Participants

This is a cross-sectional study performed via an online survey run from April 1 to April 8, 2020. The survey period corresponded to the pandemic mitigation period after prevalent peak stage of COVID-19 outbreak in China. We distributed self-administered questionnaires to ICU healthcare workers through an APP called Wenjuanxing (www.wjx.cn).

Before filling in the questionnaires, the respondents were required to fill in the informed consent form, promising that they were ICU medical workers in China and filled in the questionnaires voluntarily.

### Questionnaires

The nine-item Patient Health Questionnaire (PHQ-9) and seven-item General Anxiety Disorder questionnaire (GAD-7) were used to assess depressive and anxiety symptoms. Meanwhile, basic demographic information including gender, age, occupation, education, ICU work time, hospital level, marriage, numbers of elderlies and children, vacation days, night duty number, frontline or second-line anti-pandemic work, labor, and income variation were also collected. Frontline anti-pandemic work was defined as contact with confirmed COVID-19 infections; in contrast, second-line was defined as no contact.

### Mental Health Assessment

The PHQ-9 scale reflected symptom of depression, which consisted of nine questions each scored 0–3 according to respondents' answer. The final PHQ-9 total scores are categorized as follows: no depression (0–4), mild depression (5–9), moderate depression (10–14), and severe depression (15–27). Similarly, the GAD-7 scale reflected symptom of anxiety, which consisted of seven questions each scored 0–3. The final total scores are also categorized as follows: no anxiety (0–4), mild anxiety (5–9), moderate anxiety (10–14), and severe anxiety (15–21).

### Statistical Analysis

Data analysis was performed using SPSS version 22.0 (SPSS Inc., Chicago, IL, USA). For count data, frequencies and percentages were used, and the chi-square test was used to compare the data for different categorical variables. Non-normalized distributed parameters were expressed as median (interquartile range), Mann–Whitney *U*-test was used to compare non-normalized distributed parameters. A *P*-value <0.05 was considered statistically significant. Multivariable logistic regression analysis was carried out to evaluate the association between mental health status and potential predictors, and variables were determined to contribute to the model if the significance level for the Wald inclusion test statistic was <0.05.

## Results

### Basic Demographic Characteristics

There were 731 ICU healthcare workers who were finally enrolled in our study, including 325 (44.5%) frontline and 406 (55.5%) second-line ICU healthcare workers. In all, 303 (41.5%) were male, 383 (52.4%) were doctors, 617 (84.4%) aged 26–45 years, 278 (38.0%) worked in teaching hospital, 570 (78.0%) had worked in current occupation for more than 5 years, 381 (52.1%) had night duty ≥10, and 449 (61.5%) experienced income reduction during the COVID-19 pandemic. ICU healthcare workers in the frontline were older (*P* < 0.001), higher educated (*P* < 0.001), higher paid (*P* < 0.001), had more working experience (*P* < 0.001), and more vacation days (*P* < 0.001); there were also more males (*p* < 0.001) and doctors (*p* < 0.001) in frontline group. Detailed basic demographic characteristics are shown in Table 1.

**Table 1:** Basic demographic characteristics of health workers enrolled in the study.

**Characteristics**	**Frontline**	**Second-line**	**Total**	***P*-value**
	**(*n* = 325)**	**(*n* = 406)**	**(*n* = 731)**	
**Gender**				<0.001
Male	167 (51.4)	136 (33.5)	303 (41.5)	
Female	158 (48.6)	270 (66.5)	428 (58.5)	
**Age, years**				<0.001
≤ 25	13 (4.0)	36 (8.9)	49 (6.7)	
26–45	271 (83.4)	346 (85.2)	617 (84.4)	
>45	41 (12.6)	24 (5.9)	65 (8.9)	
**Occupation**				<0.001
Doctor	194 (59.7)	189 (46.6)	383 (52.4)	
Nurse	131 (40.3)	217 (53.4)	348 (47.6)	
**Education**				<0.001
≤ Undergraduate	234 (72.0)	338 (83.2)	572 (78.2)	
≥Postgraduate	91 (28.0)	68 (16.8)	159 (21.8)	
**ICU work time, years**				<0.001
≤ 5	47 (14.5)	114 (28.1)	161 (22.0)	
>5	278 (85.5)	292 (71.9)	570 (78.0)	
**Hospital level**				0.08
Teaching hospital	135 (41.5)	143 (35.2)	278 (38.0)	
Non-teaching hospital	190 (58.5)	263 (64.8)	453 (62.0)	
**Marriage**				0.31
Married	266 (81.8)	320 (78.8)	586 (80.2)	
Unmarried or divorce	59 (18.2)	86 (21.2)	145 (19.8)	
**Children need support**				0.12
0	80 (24.6)	121 (29.8)	201 (27.5)	
≥1	245 (75.4)	285 (70.2)	530 (72.5)	
**Elderlys need support**				0.76
≤ 2	139 (42.8)	169 (41.6)	308 (42.1)	
≥3	186 (57.2)	237 (58.4)	423 (57.9)	
**Vacation, days**				0.001
0	111 (34.2)	186 (45.8)	297 (40.6)	
1–6	118 (36.3)	102 (25.1)	220 (30.1)	
≥7	96 (29.5)	118 (29.1)	214 (29.3)	
**Night duty number**				0.41
≤ 3	75 (23.1)	101 (24.9)	176 (24.1)	
4–9	85 (26.1)	89 (21.9)	174 (23.8)	
≥10	165 (50.8)	216 (53.2)	381 (52.1)	
**Labor**				0.76
Unchanged	119 (36.6)	157 (38.6)	276 (37.8)	
Increased	12 (3.7)	12 (3.0)	24 (3.3)	
Reduced	194 (59.7)	237 (58.4)	431 (58.9)	
**Income**				<0.001
Unchanged	98 (25.4)	129 (31.8)	227 (31.0)	
Increased	49 (37.3)	6 (1.5)	55 (7.5)	
Reduced	178 (37.3)	271 (66.7)	449 (61.5)	

*Data were expressed as no. %*.

### Mental Health Status

The severity of depressive and anxiety symptoms were categorized according to the PHQ-9 and GAD-7 scores. In this study, only 249 (34.1%) and 302 (41.3%) ICU healthcare workers were finally recorded to be free from depressive and anxiety symptoms, respectively. The rest of the ICU healthcare workers suffered from different degrees of depressive and anxiety symptoms. We further compared mental health status between frontline and second-line ICU healthcare workers, and no significant difference was found in the severity of depressive (*P* = 0.15) and anxiety (*P* = 0.56) symptoms. Detailed mental health information are shown in Table 2.

**Table 2:** Detailed mental health information.

**Mental health**	**Frontline**	**Second-line**	**Total**	***P*-value**
	**(*n* = 325)**	**(*n* = 406)**	**(*n* = 731)**	
**Depression**				
NO	119 (36.6)	130 (32.0)	249 (34.1)	
Mild	105 (32.3)	165 (40.6)	270 (36.9)	0.15
Moderate	59 (18.2)	64 (15.8)	123 (16.8)	
Severe	42 (12.9)	47 (11.6)	89 (12.2)	
**Anxiety**				
NO	132 (40.6)	170 (41.9)	302 (41.3)	
Mild	134 (41.2)	178 (43.8)	312 (42.7)	0.56
Moderate	44 (13.6)	43 (10.6)	87 (11.9)	
Severe	15 (4.6)	15 (3.7)	30 (4.1)	

*Data were expressed as no. %*.

### Influencing Factors of Mental Health

**Figure 1:**
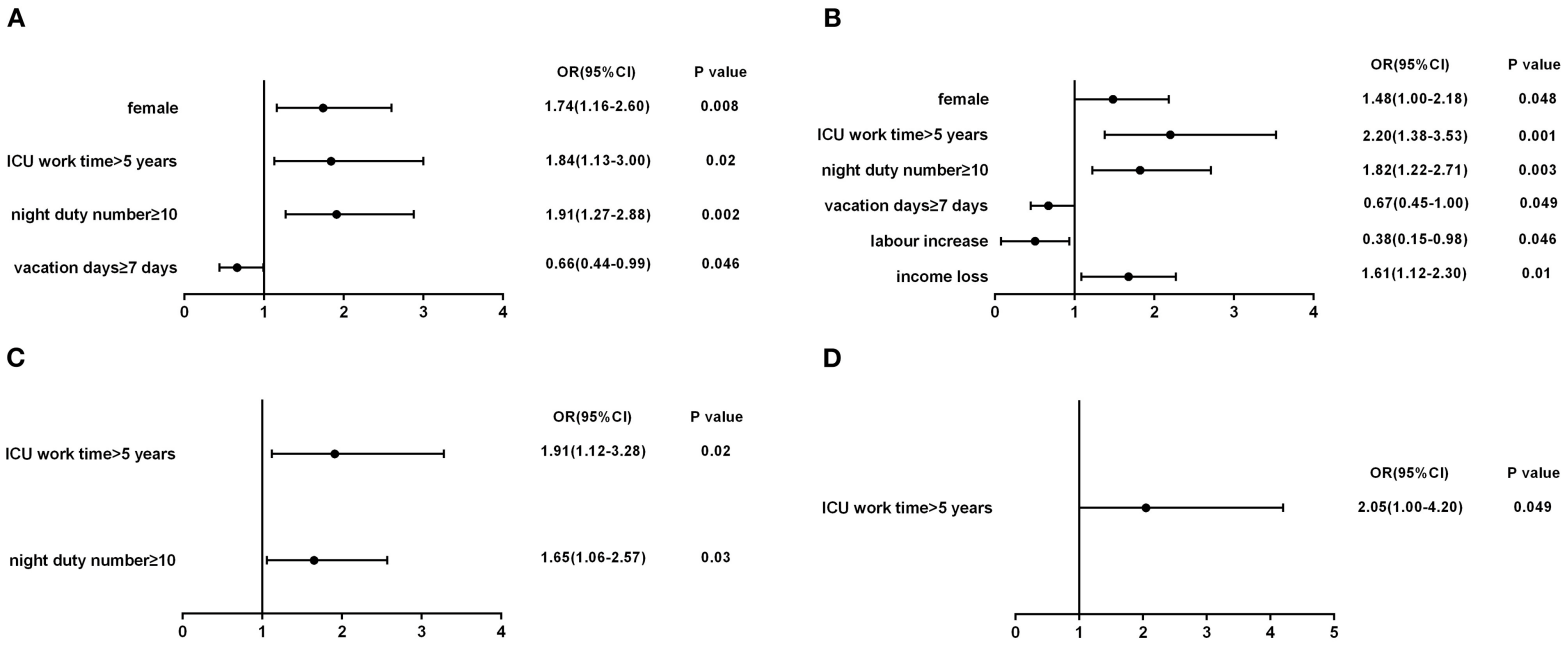
**(A)** Influencing factors of depression; **(B)** influencing factors of anxiety; **(C)** risk factors of moderate-severe depression; **(D)** risk factor of moderate-severe anxiety.

Four factors had been identified as being significantly associated with depression symptoms (Figure 1A): being female (OR, 1.74; 95% CI, 1.16–2.60; *P* = 0.008), ICU work time >5 years (OR, 1.84; 95% CI, 1.13–3.00; *P* = 0.02), vacation days ≥7 days (OR, 0.66; 95% CI, 0.44–0.99; *P* = 0.046), and night duty number ≥10 (OR, 1.91; 95% CI, 1.27–2.88; *P* = 0.002). We also identified six factors as being significantly associated with anxiety symptoms (Figure 1B): being female (OR, 1.48; 95% CI, 1.00–2.18; *P* = 0.048), ICU work time >5 years (OR, 2.20; 95% CI, 1.38–3.53; *P* = 0.001), vacation days ≥7 days (OR, 0.67; 95% CI, 0.45–1.00; *P* = 0.049), night duty number ≥10 (OR, 1.82; 95%CI, 1.22–2.71; *P* = 0.003), labor increase (OR, 0.38; 95% CI, 0.15–0.98; *P* = 0.046), and income loss (OR, 1.61; 95% CI, 1.12–2.30; *P* = 0.01). Additionally, we found ICU work time >5 years (OR, 1.91; 95% CI, 1.12–3.28; *P* = 0.02) and night duty number ≥10 (OR, 1.65; 95% CI, 1.06–2.57; *P* = 0.03) were risk factors of developing moderate–severe depressive symptoms (Figure 1C). ICU work time >5 years also helped to develop moderate–severe anxiety symptoms (OR, 2.05; 95% CI, 1.00–4.20; *P* = 0.049) (Figure 1D).

## Discussion

On May 13th, the UN secretary general António Guterres stressed the adverse impact of COVID-19 pandemic on people's mental health, especially health care workers. Among all medical departments, ICU was responsible for the most critical COVID-19 cases, which might bring more mental burden to ICU healthcare workers. This is the first mental health study that examined the psychological impact of COVID-19 pandemic on ICU healthcare workers in China.

Our study revealed a high prevalence of mental disorders among 731 ICU healthcare workers; 65.9 and 58.7% respondents were finally screened symptoms of depression and anxiety, respectively, which were consistent with previous studies (13, 14). However, no significant mental health difference had been found between frontline and second-line ICU healthcare workers in our study. Neither did logistic regression identify frontline work as a risk factor of developing mental disorders. This was quite different from the experience of battling the SARS pandemic, which proved that medical workers who were exposed to SARS were associated with more mental health problems (15, 16). Moreover, Zhou's study also came to the distinct conclusion that frontline medical staff were more easily to suffer from psychological disturbances during the COVID-19 pandemic (17). We think our results could be explained as follows: firstly, frontline ICU healthcare workers in our study were older, higher educated, and there were also more males and doctors in the frontline group. Previously published studies had shown that male, doctors, older age, and higher education level were negatively associated with mental health problems among medical workers (14, 18–23). Additionally, ICU healthcare workers in the frontline were higher paid due to government's financial compensation and had more vacation days, which might help to relieve their mental distress.

Secondly, our study was performed from April 1, 2020 to April 8, 2020, when medical workers obtained enough knowledge about the virus and enough medical supplies especially personal protective equipment in China. According to Maunder's studies, confidence in protective measures could be positive to keep mental health (24). Skoda's study in German also exhibited a similar conclusion that more knowledge about COVID-19 will contribute to reduce psychological burden from the pandemic (23).

Besides, our study showed that being female, longer ICU work time, and more night duty were risk factors of developing depressive and anxiety symptoms, while more vacation days could be protective. Income reduction was separately identified as risk factor of anxiety symptoms. Our results reemphasized the damage of high workload on mental health of medical staff as Zhou's study pointed out (17). To be noted, work experience was generally considered to be beneficial to mental health when treating with emergency public health incidence (25); in our study, longer ICU work time was unexpectedly identified detrimental to maintain mental health. This could be explained by the high prevalence of occupational burnout among ICU healthcare workers especially for those who had worked long years in current occupation, since occupational burnout had been proved to be associated with depressive and anxiety symptoms (26–30). This result raised concern about occupational burnout among ICU healthcare workers with long years of working.

## Conclusions

Our study had shown a high prevalence of depressive and anxiety symptoms among ICU healthcare workers. Frontline ICU work was not associated with higher risk of depressive and anxiety symptoms during the COVID-19 pandemic remission period in China. Actions like controlling night duty number, ensuring vacation, and increasing income should be taken to relieve mental health problem. Furthermore, we should pay more attention to those who had worked long years in ICU departments.

## Limitations

Following limitations should be considered in our study. Data were obtained via anonymous, self-reported questionnaires online, and the sample size enrolled was relatively small, which would inevitably bring potential selection bias. The risk of COVID-19-related symptoms of depression and anxiety may be influenced by occupational burnout among ICU healthcare workers. Finally, the study only reflected mental health status during the short survey time when China broke through the extreme stress phase of the COVID-19 pandemic.

## Data Availability Statement

The original contributions presented in the study are included in the article/supplementary material, further inquiries can be directed to the corresponding author/s.

## Ethics Statement

Ethical review and approval was not required for the study on human participants in accordance with the local legislation and institutional requirements. Written informed consent for participation was not required for this study in accordance with the national legislation and the institutional requirements.

## Author Contributions

XP designed the questionnaire, analyzed the data, and drafted the manuscript. XMe, LL, WL, CH, ZL, and XMa analyzed the data and revised the manuscript. ZZ, ZX, and BL designed the questionnaire and revised the manuscript. DX and LZ revised the manuscript. MP designed the study, analyzed the data, and revised the manuscript. All authors contributed to the article and approved the submitted version.

## Conflict of Interest

The authors declare that the research was conducted in the absence of any commercial or financial relationships that could be construed as a potential conflict of interest.
